# Target validation: Weak selectivity of LY341495 for mGluR2 over mGluR4 makes glutamate a less selective agonist

**DOI:** 10.1002/prp2.471

**Published:** 2019-05-03

**Authors:** Tyler W. McCullock, Paul J. Kammermeier

**Affiliations:** ^1^ Department of Pharmacology and Physiology University of Rochester Medical Center Rochester New York

**Keywords:** antagonist, calcium channel, GPCR, homodimer, LY341495, patch‐clamp

## Abstract

Metabotropic glutamate receptors (mGluRs) are class C G protein coupled receptors with widespread expression in the central nervous system. There are eight mGluRs in the mammalian genome. Research on mGluRs relies on the availability of selective compounds. While many selective allosteric compounds have been described, selectivity of orthosteric agonists and antagonists has been more difficult due to the similarity of the glutamate binding pocket across the mGluR family. LY341495 has been used for decades as a potent and selective group II mGluR antagonist. The selectivity of LY341495 was investigated here between mGluR2, a group II mGluR, and mGluR4, a group III receptor, heterologously expressed in adult rat sympathetic neurons from the superior cervical ganglion (SCG), which provides a null‐mGluR background upon which mGluRs were examined in isolation. The compound does in fact selectively inhibit mGluR2 over mGluR4, but in such a way that it makes signaling of the two receptors more difficult to distinguish. The glutamate potency of mGluR2 is about 10‐fold higher than mGluR4. 50 nmol L^−1^ LY341495 did not alter mGluR4 signaling but shifted the mGluR2 glutamate dose‐response about 10‐fold, such that it overlapped more closely with that of mGluR4. Increasing the LY341494 dose to 500 nmol L^−1^ further shifted the glutamate dose‐response of mGluR2 by another ~10‐fold, but also shifted that of mGluR4 similarly. Thus, while glutamate is a moderately selective agonist of mGluR2 over mGluR4 when applied alone, in the presence of increasing concentrations of LY341495, this selectivity of glutamate is lost.

**Abbreviations**
GPCRs: protein coupled receptors
mGluRs: Metabotropic glutamate receptors
SCG: superior cervical ganglion
UCAR: University Committee on Animal Resources
Vcommand: command voltage

## 1 INTRODUCTION

The mammalian metabotropic glutamate receptor (mGluR) family is comprised of eight genes encoding class C G protein coupled receptors (GPCRs) with abundant expression throughout the central and peripheral nervous systems.^1^ Due to this widespread expression, mGluRs participate in several physiological and pathophysiological processes such as learning and memory,^2^, ^3^ modulation of neurotransmitter release, and have been implicated as therapeutic targets in pathologies such as epilepsy, schizophrenia, Parkinson's Disease, and others.^4^, ^5^, ^6^

Like other class C GPCRs, mGluRs are obligate dimers.^7^, ^8^ Each member of the mGluR family forms disulfide linked homodimers and some combinations of mGluR subtypes can heterodimerize.^9^, ^10^, ^11^ Interestingly, the dimerization state of these receptors can have profound effects on their sensitivity to selective pharmacological compounds.^10^, ^11^, ^12^ For these reasons, the need for selective compounds is critical for driving research into understanding how mGluRs participate in physiological processes. For these studies to be informative, we must also be aware of whether and how each compound functions on unintended targets. The selective group II mGluR competitive antagonist LY341495 13, 14 has been used extensively for two decades, in many cases to diagnose the identity of mGluRs performing various physiological functions. Here, we examine the effect of LY341495 inhibition of mGluR2 and mGluR4 signaling, group II and III mGluRs, respectively. We find that paradoxically, despite the selectivity of the compound for mGluR2 signaling, its application may lead to effects of these receptors being more difficult to distinguish.

## 2 MATERIALS AND METHODS

### 2.1 Cell isolation, cDNA injection and molecular biology

Animal protocols were approved by the university committee on animal resources (UCAR). Briefly, SCGs were removed from adult male Wistar rats (175‐225 g) after CO_2_ euthanasia and decapitation, and incubated in Earle's balanced salt solution (InVitrogen, Life Technologies Carlsbad, CA) containing 0.5 mg/mL trypsin (Worthington Biochemicals, Freehold, NJ) & 0.7 mg/mL collagenase D (Roche Life Science, Penzburg, Germany) for 60 minutes at 35°C. The cells were transferred to minimum essential medium (InVitrogen/Gibco), plated on poly‐l‐lysine (Sigma Chemical Co., St. Louis, MO) coated tissue culture dishes and incubated at 37°C for 2‐4 hours before cDNA injection. Cells were incubated 12‐20 hours at 37°C (95% air and 5% CO_2_; 100% humidity) and electrophysiology experiments were performed the next day.

Injection of cDNA was performed with an Eppendorf 5247 microinjector and InjectMan NI 2 micromanipulator (Madison, WI) 3‐5 hours following cell isolation. Injection electrodes were made with a Sutter P‐97 horizontal electrode puller (Novato, CA) from thin–walled, borosilicate glass (World Precision Instruments, Sarasota, FL). Plasmids were stored at −20°C as a 1 μg/μL stock solution in TE buffer (10 mmol L^−1^ TRIS, 1 mmol L^−1^ EDTA, pH 8). Rat mGluR2 (pCI) and human 4 (pCDNA3.1) constructs were injected at 50 and 150 ng μL^−1^, respectively. Cells were coinjected with EGFP, EYFP, or mNeonGreen cDNA (0.02 μg/μL; pmNeonGreen‐N1; Allele Biotechnology, San Diego, CA) for identification of successfully injected cells.

All constructs were sequence confirmed (Eurofins Genomics, Ebersburg, Germany). Midipreps were prepared using Zymo Research anion exchange columns (Irvine, CA), and amplified in either Top10 or DH5α *Escherichia coli*. (InVitrogen).

### 2.2 Electrophysiology

Patch–clamp pipettes were made with a Sutter P‐97 horizontal puller from 8250 glass (Garner Glass, Claremont, CA) and had series resistances of 1‐5 MΩ prior to electronic compensation of 80%. Whole‐cell patch‐clamp recordings were made with an Axopatch 1C or 200B patch clamp amplifier (Axon Instruments, now Molecular Devices, Sunnydale, CA). Voltage protocol generation and data acquisition were performed using custom software (courtesy Stephen R. Ikeda, NIAAA, Rockville, MD) on a Macintosh G4 computer (Apple Computer, Cupertino, CA) with an InstruTech (Port Washington, NY; now HEKA Elektronik) ITC‐18 data acquisition board. Currents were low–pass filtered at 3 kHz using the 4‐pole Bessel filter in the patch clamp amplifiers, digitized at 2‐5 kHz and stored on the computer for later analysis. Experiments were performed at 21‐24°C (room temperature). Patch–clamp data analysis was performed using the Igor Pro software package (Wavemetrics, Lake Oswego, OR).

The external (bath) recording solution consisted of: 155 mmol L^−1^ tris hydroxymethyl aminomethane, 20 mmol L^−1^ 4‐(2‐Hydroxyethyl)‐1‐piperazineethanesulfonic acid (HEPES), 10 mmol L^−1^ glucose, 10 mmol L^−1^ CaCl_2_, and 0.3 μmol L^−1^ tetrodotoxin (TTX), pH 7.4. The internal (pipette) solution contained: 120 mmol L^−1^ N‐methyl‐d‐glucamine (NMG) methanesulfonate, 20 mmol L^−1^ TEA, 11 mmol L^−1^ EGTA, 10 mmol L^−1^ HEPES, 10 mmol L^−1^ sucrose, 0.1 μmol L^−1^ CaCl_2_, 4 MgATP, 0.3 mmol L^−1^ Na_2_GTP, and 14 mmol L^−1^ tris creatine phosphate, pH 7.2. l‐Glutamate (Sigma) was used as the agonist for mGluRs. LY341495 was obtained from Tocris Bioscience (R&D Systems, Minneapolis, MN). Solutions were applied to cells with a custom, gravity–driven perfusion system positioned ~100 μm from the cell, allowing rapid solution exchange (≤250 milliseconds).

## 3 RESULTS

### 3.1 Low concentrations of LY341495 selectively inhibit mGluR2

**Figure 1:**
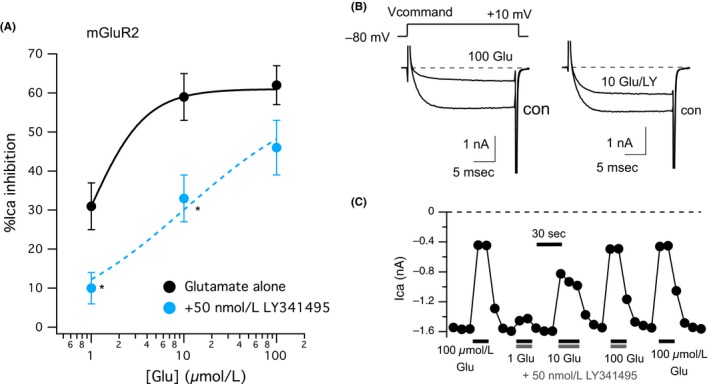
Inhibition of mGluR2 signaling by 50 nmol L^−1^ LY341495. A, Glutamate dose‐response curve for mGluR2 expressed in rat SCG neurons using inhibition of voltage gated calcium channels as an assay for receptor signaling. Responses are expressed as % inhibition of the whole–cell calcium currents in primary cultures of SCG neurons. Responses to 1, 10, and 100 μmol L^−1^ glutamate in SCG neurons expressing mGluR2 are shown in black circles, reflecting inhibitions of 31% ± 6%, 59% ± 6%, and 62% ± 5%, respectively, n = 8. In the presence of 50 nmol L^−1^ LY341495, 1, 10, and 100 μmol L^−1^ glutamate inhibited the currents by 10% ± 4%, 33% ± 6%, and 46% ± 7%, respectively, n = 7 (light blue). Lines show fits to the Hill equation with the baseline fixed to 0 and the maximum effect fixed to 62%. B, Sample current traces from an mGluR2 expressing SCG neuron before and during application of 100 μmol L^−1^ glutamate alone (“con” and “100 Glu”; left) or 10 μmol L^−1^ glutamate with 50 nmol L^−1^ LY341495 (“con” and “10 Glu/LY”; right). Scale bars for current traces are shown as insets. Voltage protocols used to elicit currents are shown (left, above), and consist of 25 milliseconds steps to +10 mV every 10 seconds. C, Current amplitude time course for the cell illustrated in (B) showing responses to application of 100 μmol L^−1^ glutamate alone, and 1, 10, and 100 μmol L^−1^ glutamate with 50 nmol L^−1^ LY341495, as indicated. Current amplitudes were measured as the average current measured from 9 to 11 milliseconds following the start of each test pulse to +10 mV

As an assay for mGluR signaling, we examined the Gβγ–mediated inhibition of the voltage gated calcium channels (mainly Ca_V_2.2 and some Ca_V_2.3^15^) natively expressed in primary cultures of adult rat SCG neurons. Because these neurons provide a null background for mGluR expression,^16^ the native agonist glutamate can be used to activate the receptors in this system when made to heterologously express via intranuclear cDNA injection.^17^ As described previously,^10^, ^18^ when mGluR2 is expressed in SCG neurons, application of a range of glutamate concentrations results in strong, reversible inhibition of the calcium currents, with an EC_50_ in the low micromolar range (Figure 1A, black circles). To begin to assess the effect of the mGluR2 antagonist LY341495, we also generated a partial glutamate dose‐response curve in the presence of 50 nmol L^−1^ LY341495 (Figure 1A, light blue circles). As expected for a competitive antagonist, LY341495 shifted the effect of glutamate about 10‐fold. Figure 1B (upper inset) shows that the voltage protocol used to sample the calcium currents was a 25 milliseconds step from the holding potential of −80 mV to +10 mV, which is near the peak of the calcium current IV curve under these recording conditions. Sample control and glutamate–inhibited currents are also shown in Figure 1B in the absence (left) and presence (right) of 50 nmol L^−1^ LY341495. The current amplitude time course from the same cell is shown in Figure 1C, in which 100 μmol L^−1^ glutamate was applied alone, and a range of [glutamate] was applied in the presence of 50 nmol L^−1^ LY341495, as shown. These data are consistent with LY341495 acting as a potent, competitive antagonist at mGluR2.

**Figure 2:**
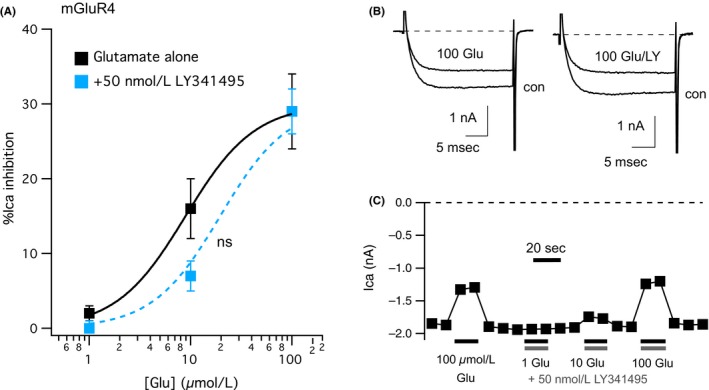
Effect on mGluR4 signaling of 50 nmol L^−1^ LY341495. A, Glutamate dose‐response curve for mGluR4, as in Figure 1. Responses to 1, 10, and 100 μmol L^−1^ glutamate in SCG neurons expressing mGluR4 are shown in black squares, reflecting inhibitions of 2% ± 1%, 16% ± 4%, and 29% ± 5%, respectively, n = 11, 10, and 10, respectively. In the presence of 50 nmol L^−1^ LY341495, 1, 10, and 100 μmol L^−1^ glutamate inhibited the currents by 0% ± 1%, 7% ± 2%, and 29% ± 3%, respectively, n = 3, 4, and 4, respectively (light blue), which are not significantly different from control (*P* < 0.05, *t*‐test). B, Sample current traces from an mGluR4 expressing SCG neuron before and during application of 100 μmol L^−1^ glutamate alone (“con” and “100 Glu”; left) or 100 μmol L^−1^ glutamate with 50 nmol L^−1^ LY341495 (“con” and “100 Glu/LY”; right). Scale bars for current traces are shown as insets. C, Current amplitude time course for the cell illustrated in (B) showing responses to application of 100 μmol L^−1^ glutamate alone, and 1, 10, and 100 μmol L^−1^ glutamate with 50 nmol L^−1^ LY341495, as indicated. Current amplitudes were measured as in Figure 1C

Next, to examine selectivity of the effect of LY341495, we examined its effects on mGluR4 signaling in SCG neurons (Figure 2). Consistent with previous data in this system,^10^ glutamate was a less potent and less efficacious agonist of mGluR4 than at mGluR2. It should be noted that we cannot rule out differences in receptor expression levels contributing to these differences, but the relative potency of glutamate for these receptors is consistent with reported values.^19^ Although the glutamate response is not clearly saturating in Figure 2A, previous data from the same system indicated that the effect of 100 μmol L^−1^ glutamate is saturating, and has a maximal response of 25%‐30% inhibition in this system^10^ (Figure 2A, black squares). As expected, application of 50 nmol L^−1^ LY341495 with 1‐100 μmol L^−1^ glutamate did not produce a significant reduction in the magnitude of calcium current inhibition through mGluR4 (Figure 2A, light blue squares). Figure 2B and C show sample current traces and a current amplitude time course of a cell upon application of 100 μmol L^−1^ glutamate alone or a range of glutamate concentrations coapplied with 50 nmol L^−1^ LY341495. Together, these data are consistent with LY341495 acting as a selective antagonist of mGluR2 when applied at 50 nmol L^−1^.

### 3.2 Higher concentrations of LY341495 inhibit mGluR4

**Figure 3:**
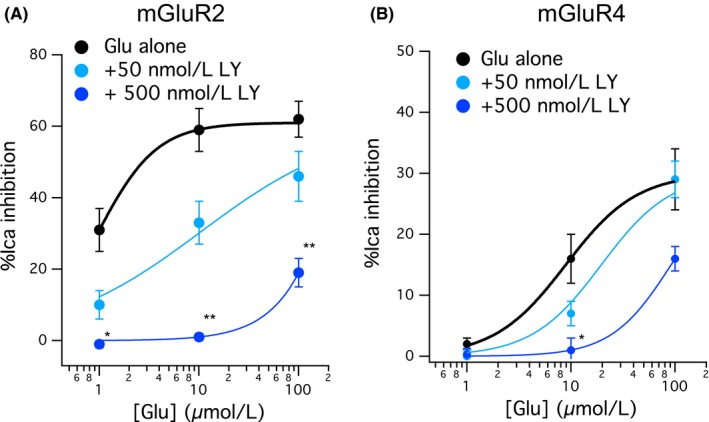
Effect of 50 and 500 nmol L^−1^ LY341495 on mGluR2 and mGluR4. A, Glutamate dose‐response curves from mGluR2 expressing SCG neurons with glutamate alone (black circles), glutamate with 50 nmol L^−1^ LY341495 (light blue) and 500 nmol L^−1^ LY341495 (dark blue). B, Glutamate dose‐response curves from mGluR4 expressing SCG neurons with glutamate alone (black circles), glutamate with 50 nmol L^−1^ LY341495 (light blue) and 500 nmol L^−1^ LY341495 (dark blue). * indicates significantly different (*P* < 0.05) from control, ** indicates significant difference from both the control and the 50 nmol L^−1^ LY condition (one‐way ANOVA)

As described above, 50 nmol L^−1^ LY341495 selectively inhibited mGluR2 signaling and did not inhibit mGluR4. However, because glutamate acts on mGluR2 with higher potency than mGluR4, this resulted in glutamate activating the two receptors with less selectivity. To determine if increasing doses of LY341495 could more completely inhibit mGluR2 signaling, the effect of 500 nmol L^−1^ LY341495 was examined. As shown in Figure 3A, 500 nmol L^−1^ LY341495 did in fact inhibit mGluR2 more strongly than 50 nmol L^−1^, resulting in an approximately 100‐fold shift of the glutamate dose‐response curve compared to control (Figure 3A, dark blue). The results from the experiments in Figure 1 are also shown for comparison (black and light blue curves). The effect of 500 nmol L^−1^ LY341495 was also tested on SCG neurons expressing mGluR4 (Figure 3B). Unfortunately, at this higher dose LY341495 also produced a significant inhibition of the mGluR4 glutamate response, shifting it about 10‐fold to the right (Figure 3B, dark blue).

**Figure 4:**
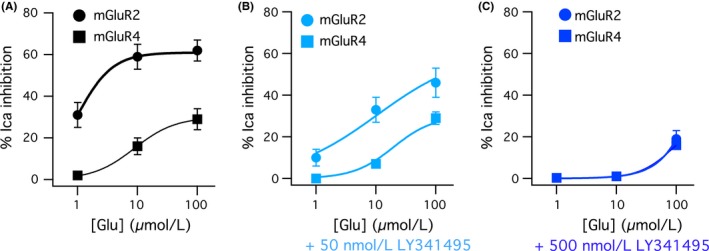
Increasing concentrations of LY341495 cause the glutamate dose‐response curves of mGluR2 and mGluR4 to converge. Data are from previous Figures A, Glutamate dose‐response curves for mGluR2 (circles) and mGluR4 (squares) in the absence of LY341495. B, Glutamate dose‐response curves for mGluR2 (circles) and mGluR4 (squares) in the presence of 50 nmol L^−1^ LY341495. C, Glutamate dose‐response curves for mGluR2 (circles) and mGluR4 (squares) in the presence of 500 nmol L^−1^ LY341495

To assess the net effect of LY341495 on signaling of both mGluR2 and mGluR4, the glutamate dose‐response curves for both receptors are shown in Figure 4 in the absence of LY341495 (Figure 4A), and in the presence of 50 nmol L^−1^ (Figure 4B), and 500 nmol L^−1^ (Figure 4C) LY341495. These data demonstrate that due to the difference in efficacy and potency of these two receptors, glutamate alone is a fairly good discriminator of mGluR2 and mGluR4 signaling (Figure 4A). However, while LY341495 is in fact a selective inhibitor of mGluR2 over mGluR4 signaling, its application actually makes it more difficult to discriminate the two receptors based on the glutamate responses. In the presence of 50 nmol L^−1^ LY341495, mGluR2 but not mGluR4 signaling is inhibited, but this results in the apparent potency of mGluR2 to shift closer to that of mGluR4. Further, in the presence of 500 nmol L^−1^ LY341495, the apparent potencies of both receptors shift such that responses to the two receptors are indistinguishable (Figure 4C).

### 3.3 Inhibition of mGluR2/4 heterodimers by LY341495

**Figure 5:**
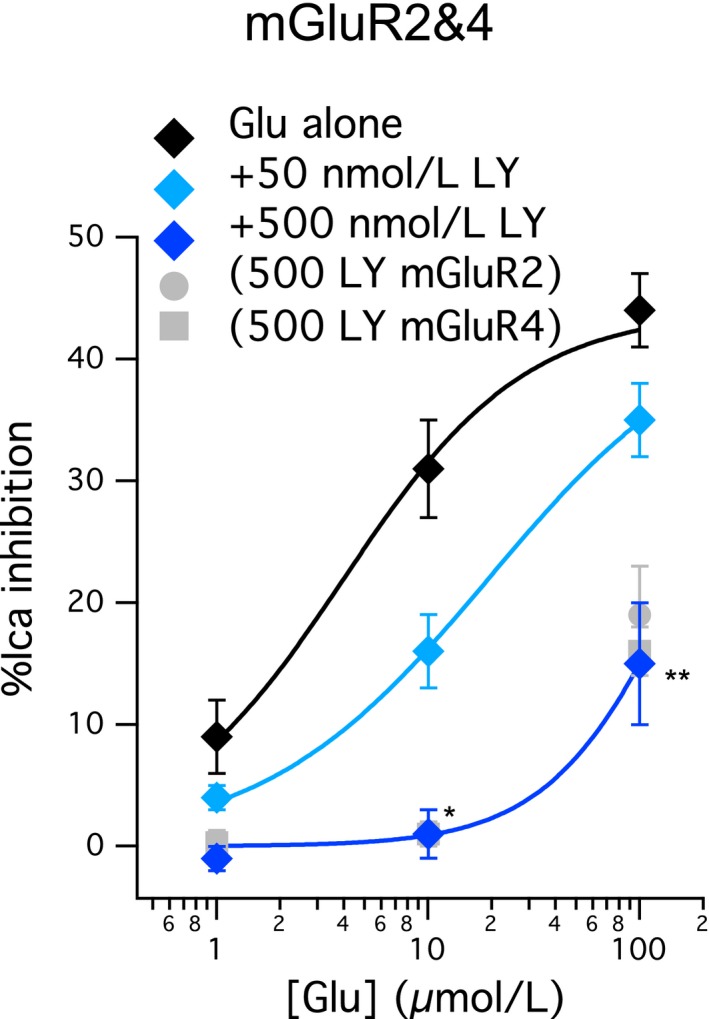
Effect of LY341495 on mGluR2/4 heterodimers. Glutamate dose‐response curves for mGluR2/4, as in Figure 1. Responses to 1, 10, and 100 μmol L^−1^ glutamate in SCG neurons expressing mGluR2/4 are shown in black diamonds, reflecting inhibitions of 9% ± 3%, 32% ± 4%, and 44% ± 3%, respectively, n = 15. In the presence of 50 nmol L^−1^ LY341495, 1, 10, and 100 μmol L^−1^ glutamate inhibited the currents by 4% ± 1%, 16% ± 3%, and 35% ± 3%, respectively, n = 5 (light blue). In the presence of 500 nmol L^−1^ LY341495, 1, 10, and 100 μmol L^−1^ glutamate inhibited the currents by 0% ± 1%, 1 ± 2%, and 15% ± 5%, n = 6, 6, and 7, respectively (dark blue). * indicates significantly different (*P* < 0.05) from control, ** indicates significant difference from both the control and the 50 nmol L^−1^ LY condition (one‐way ANOVA)

Previously we have shown that coexpression of mGluR2 and mGluR4 in rat SCG neurons leads to preferential formation of mGluR2/4 heterodimers.^10^ Heterodimerization of these receptors has been reported in heterologous expression systems^9^ and with natively expressed receptors in the prefrontal cortex.^11^ To determine the effects of LY341495 on mGluR2/4 heterodimers, the two receptors were expressed in SCG neurons as in the previous study,^10^ and their activity as assessed as in Figures 1 and 2. Figure 5 shows a partial glutamate dose‐response curve for mGluR2/4 that is consistent with our data reported previously, with potency and efficacy both intermediate to that observed with each receptor expressed alone.^10^ Application of 50 nmol L^−1^ LY341495 produced a significant reduction in the response of 10 μmol L^−1^ glutamate and an apparent shift of the glutamate dose‐response curve by approximately 5‐fold (Figure 5, light blue). Increasing the concentration of LY341495 to 500 nmol L^−1^ produced a more pronounced shift in the curve (Figure 5, dark blue), similar to its effects on both mGluR2 and mGluR4 (see Figure 4). Indeed, in the presence of 500 nmol L^−1^ LY341495, the responses of mGluR2, mGluR4, and mGluR2/4 to 1, 10, and 100 μmol L^−1^ glutamate were indistinguishable (Figure 5, gray circles and squares, included for comparison).

Together these data highlight a surprising scenario in which an mGluR2 selective inhibitor actually creates a situation in which signaling of mGluR2, mGluR4, and the mGluR2/4 heterodimer becomes more difficult to discern.

## 4 DISCUSSION

The mGluR2 selective, competitive antagonist LY341495^2^, ^13^ has been used in numerous studies as an indication of the participation of mGluR2 or group II mGluRs generally.^20^, ^21^, ^22^ We wished to evaluate some specific aspects of the selectivity of this compound in primary cultures of rat SCG neurons, a neural system in which mGluR subtypes could express in clear isolation.^16^ Initially, this was motivated by a project in which we aimed to examine the pharmacological profile of mGluR2/4 heterodimers as a distinct entity from either mGluR2 or mGluR4 homodimers.^10^ For that study, we hoped that the compound LY341495, a nominally mGluR2 selective antagonist, could dramatically shift the glutamate dose‐response curve of mGluR2 without affecting that of mGluR4 when the receptors were expressed separately (as homodimers). As the data above demonstrate, the compound was not useful for that purpose as it has only a narrow concentration window in which it specifically inhibits mGluR2 signaling over mGluR4. Further, in this range (~50 nmol L^−1^), the mGluR2 response is shifted only slightly. However, due to the fact that glutamate activates mGluR2 with slightly higher potency than mGluR4 in the absence of inhibitors, this had the unfortunate effect of causing the responses to glutamate of the two receptors to converge. Further, when the concentration of LY341495 was increased to 500 nmol L^−1^, this convergence was even more pronounced such that the responses of the two receptors to glutamate were virtually indistinguishable. These data indicate that the selectivity of LY341495 for mGluR2 over mGluR4 is not sufficient for many applications to distinguish the contributions of these receptors.

In this study, we observed an apparent 10‐fold difference in the effects of LY341495 on mGluR2 and mGluR4. However, it should be noted that this may be an underestimate of the selectivity of the compound. The reported K_d_ for LY341495 binding to mGluR2 and mGluR3 is in the low nmol L^−1^ range.^23^ In this study, we did not test concentrations of LY341495 below 50 nmol L^−1^, which may have produced some inhibition of mGluR2 signaling, so we cannot rule out that the difference in effects on mGluR2 and mGluR4 is greater than 10‐fold.

## DISCLOSURE

None declared.
